# Supplementary material for: Determinants of social participation in people with disability
Source: PLoS One. 2024 May 20;19(5):e0303911. doi: 10.1371/journal.pone.0303911 (PMC11104585; doi:10.1371/journal.pone.0303911)
Supplement: S1 Text — (PDF) [file pone.0303911.s001.pdf]

PATIENT EVALUATION FORM

NAME SURNAME:

PHONE NO:

AGE/GENDER:

MARITAL STATUS: ◯ **MARRIED** ◯ **SINGLE**

EDUCATIONAL BACKGROUND:

◯ **NOT LITERATE**

◯ **LITERATE**

◯ **PRIMARY EDUCATION**

◯ **HIGH SCHOOL**

◯ **UNIVERSITY**

OPERATING STATUS: ◯ **NOT WORKING** ◯ **WORKING**

DISABILITY TYPE AND CAUSE: ◯ **CONGENITAL** ◯ **ACQUIRED**

◯ **ORTHOPEDIC** ◯ **NEUROLOGICAL** ◯ **OTHER: …..**

WHO LIVES WITH: ◯ **WITH FAMILY** ◯ **ALONE**

NUMBER OF PEOPLE LIVING IN THE HOUSE: ◯ **1-2** ◯ **3-4** ◯ **5-7** ◯ **>8**

HOW MANY YEARS HAVE HE BEEN DISABLED: ◯ **1-5 YEARS** ◯ **6-9 YEARS** ◯ **>10 YEARS**

SOURCE OF LIVING: ◯ **OWN SALARY** ◯ **FAMILY**

MONTHLY HOUSEHOLD INCOME: ……………………………………..

MOBILITY:

◯ **WALKING INDEPENDENT**

◯ **WALKS WITH ASSISTANCE/DEVICE**

◯ **IMMOBILE**

Keele Assessment of Participation

1. During the past 4 weeks, I have moved around in my home, **<u>as and when I have wanted.</u>**
   (All of the time/ Most of the time/ Some of the time/ A little of the time/ None of the time)

2. During the past 4 weeks, I have moved around outside my home, **<u>as and when I have wanted.</u>**
   (All of the time/ Most of the time/ Some of the time/ A little of the time/ None of the time)

3. During the past 4 weeks, my self-care needs (examples are washing, toileting, dressing, feeding, maintaining health) have been met, **<u>as and when I have wanted.</u>**
   (All of the time/ Most of the time/ Some of the time/ A little of the time/ None of the time)

4. During the past 4 weeks, my home has been looked after, **<u>as and when I have wanted.</u>**
   (All of the time/ Most of the time/ Some of the time/ A little of the time/ None of the time)

5. During the past 4 weeks, my things (belongings) have been looked after, **<u>as and when I have wanted.</u>**
   (All of the time/ Most of the time/ Some of the time/ A little of the time/ None of the time)

6. Do you have any relatives, or other people, who depend on you? (Yes/No)
   If yes, during the past 4 weeks, were these people looked after, **<u>as and when you wanted?</u>**
   (All of the time/ Most of the time/ Some of the time/ A little of the time/ None of the time)

7. During the past 4 weeks, I have met and spoken to other people **<u>as and when I have wanted.</u>**
   (All of the time/ Most of the time/ Some of the time/ A little of the time/ None of the time)

8. During the past 4 weeks, I, or someone else on my behalf, have managed my money, **<u>as I have wanted.</u>**
   (All of the time/ Most of the time/ Some of the time/ A little of the time/ None of the time)

9. Do you choose to take part in paid or voluntary work? (Yes/No)
   If yes, during the past 4 weeks, have you taken part in paid or voluntary work, **<u>as and when you have wanted?</u>**
   (All of the time/ Most of the time/ Some of the time/ A little of the time/ None of the time)

10. Do you choose to take part in education or training courses? (Yes/No)
    If yes, during the past 4 weeks, have you taken part in education or training, **<u>as and when you have wanted?</u>**
    (All of the time/ Most of the time/ Some of the time/ A little of the time/ None of the time)

11. Do you choose to take part in social activities? (Yes/No)
    If yes, during the past 4 weeks, have you taken part in social activities, **<u>as and when you have wanted?</u>**
    (All of the time/ Most of the time/ Some of the time/ A little of the time/ None of the time)

Personal Wellbeing Index - Adults

1. "How satisfied are you **with your standard of living?"**

No satisfaction at all										Completely Satisfied
0	1	2	3	4	5	6	7	8	9	10
☐	☐	☐	☐	☐	☐	☐	☐	☐	☐	☐

2. "How satisfied are you **with your health?"**

No satisfaction at all										Completely Satisfied
0	1	2	3	4	5	6	7	8	9	10
☐	☐	☐	☐	☐	☐	☐	☐	☐	☐	☐

3. "How satisfied are you **with what you are achieving in life?**"

No satisfaction at all										Completely Satisfied
0	1	2	3	4	5	6	7	8	9	10
☐	☐	☐	☐	☐	☐	☐	☐	☐	☐	☐

4. "How satisfied are you **with your personal relationships?**"

No satisfaction at all										Completely Satisfied
0	1	2	3	4	5	6	7	8	9	10
☐	☐	☐	☐	☐	☐	☐	☐	☐	☐	☐

5. "How satisfied are you **with how safe you feel?**"

No satisfaction at all 0	1	2	3	4	5	6	7	8	9	Completely Satisfied 10
☐	☐	☐	☐	☐	☐	☐	☐	☐	☐	☐

6. "How satisfied are you **with feeling part of your community?**"

No satisfaction at all 0	1	2	3	4	5	6	7	8	9	Completely Satisfied 10
☐	☐	☐	☐	☐	☐	☐	☐	☐	☐	☐

7. "How satisfied are you **with your future security?**"

No satisfaction at all 0	1	2	3	4	5	6	7	8	9	Completely Satisfied 10
☐	☐	☐	☐	☐	☐	☐	☐	☐	☐	☐
