# Supplementary material for: Determinants of social participation in people with disability
Source: PLoS One. 2024 May 20;19(5):e0303911. doi: 10.1371/journal.pone.0303911 (PMC11104585; doi:10.1371/journal.pone.0303911)
Supplement: Attachment — Submitted filename: Responce to reviewers.docx [file pone.0303911.s003.docx]

**Responce to reviewers**

**Editor - inChief**

Thank you for your kind review.

The headings of the manual were edited according to PLOS ONE's style requirements.

The full ethics statement and the name of the ethics committee have been added.

**Reviewer 1**

Thank you for your kind review.

This manuscript makes a valuable contribution to the literature on disability studies and social participation. By investigating the determinants of social participation in people with disabilities, specifically focusing on mobility levels, employment status, and personal wellbeing, the study provides insights that can guide community-based rehabilitation efforts and the development of social policies. The findings emphasize the importance of addressing mobility limitations, promoting employment opportunities, and enhancing personal wellbeing to facilitate greater social participation among individuals with disabilities. Additionally, the study sheds light on gender disparities, highlighting the higher social participation levels among females, as well as the impact of neurological disabilities on social integration. Overall, this research adds depth to our understanding of the factors influencing social participation in the disabled population and underscores the need for targeted interventions and inclusive policies to improve their quality of life and societal engagement.

**Reviewer 2**

Thank you for your kind review.

1) Regarding the enrollment of research subjects, they were indeed consecutive patients who visited the Physical Medicine and Rehabilitation outpatient clinic by the Health Board for Persons with Disabilities within the specified timeframe. However, we omitted to mention the exclusion criteria in the manuscript. The exclusion criteria included individuals with mental or cognitive disabilities due to the focus on physical disabilities and their impact on social participation. We included this information in the revised manuscript to provide clarity on subject selection.

2) The types of disabilities among the research subjects were primarily physical in nature, including neurological and orthopedic disabilities. We did not include subjects with mental or cognitive disabilities, as mentioned earlier. We specified these details in the manuscript along with the definition of disability as per the World Health Organization's International Classification of Functioning, Disability, and Health (ICF).

3) The sentence regarding musculoskeletal problems indeed needs clarification. It means that since the study was conducted at a Physical Medicine and Rehabilitation outpatient clinic by the Health Board for Persons with Disabilities, it focused on individuals with physical disabilities related to musculoskeletal or neurological conditions. Purely neurological conditions without musculoskeletal involvement were not included. We rephrased this sentence to avoid confusion and provide a clearer explanation of the inclusion criteria.

4) We apologize for the errors in the text, and we ensured to correct these and provide accurate references in the revised manuscript.

5) We elaborated on the "other" types of disability in the manuscript, specifying examples and providing more information to enhance clarity.

6) The degree of disability was not assessed in this study. However, we acknowledge that assessing the severity or degree of disability could provide valuable insights, and we will consider this for future research.

7) We appreciate the suggestion to improve the discussion section regarding employment and income as significant factors. We included additional arguments and cite the suggested references to provide a more comprehensive discussion on the barriers faced by people with disabilities in the workforce and how they impact social participation.

Thank you for the constructive feedback, and we addressed these points in the revised manuscript to enhance the quality and clarity of our research.

**Reviewer 3**

1) The abstract revised to include more detail about the sample characteristics and the number of participants, ensuring that readers can better understand and interpret the results.

2) In the introduction, we expanded the theoretical framework by discussing existing knowledge on factors associated with participation, identifying gaps in the literature, and clearly justifying how this study will contribute to advancing current knowledge on the topic.

3) Regarding the methods, we acknowledge the limitations of the cross-sectional design used in the study. We reviewed the study objectives in light of these considerations and provide a clear explanation of the variables investigated, including the outcome variable and predictors. Additionally, we addressed the potential for selection bias and information bias in the discussion of methodological limitations.

4) The sample size is properly justified either through sample size calculation or based on relevant references.

5) Writing problems indicated by the word "Error!" in the results section is corrected promptly to ensure the accuracy and readability of the manuscript.

6) In the discussion section, we adopted a more critical and reflective approach to the results. This involved contrasting the findings with existing knowledge, discussing their practical implications for the field of health and rehabilitation, and highlighting whether the results were expected or surprising. We also discussed how the new findings can aid healthcare professionals in the clinical practice of assisting individuals with disabilities.

Thank you for the valuable feedback, and we incorporated these suggestions to improve the overall quality and impact of our study.
